# Patient Outcomes in Helicopter Emergency Medical Service Documentaries and on Air Ambulance Websites

**DOI:** 10.7759/cureus.53414

**Published:** 2024-02-01

**Authors:** Finlay W McMunn, Rosalyn Buckland, Rosanna E Watts, Jake Roberts, Michael D Christian

**Affiliations:** 1 Barts and the London School of Medicine and Dentistry, Queen Mary University of London, London, GBR; 2 Medicine and Surgery, Torbay and South Devon NHS Foundation Trust, Torquay, GBR; 3 Medicine and Surgery, Royal Free London NHS Foundation Trust, London, GBR; 4 Critical Care, University of British Columbia, Faculty of Medicine, Vancouver, CAN

**Keywords:** public perception, television, media, emergency, prehospital

## Abstract

Background

Helicopter emergency medical service (HEMS) documentaries attract millions of viewers, and publicly available patient stories on Air Ambulance websites are vital to raise awareness and funding for Air Ambulance charities in the United Kingdom (UK). Despite abundant research investigating how fictional programs and news outlets present patient health outcomes, there are no comprehensive studies that investigate how non-fictional HEMS documentaries or Air Ambulance websites present patient outcomes. The aim of this study is to capture the frequency of poor outcomes (mortality) in patients broadcasted on documentaries focusing on HEMS and the patient stories section of UK Air Ambulance websites.

Methods

A retrospective cohort study reviewed five HEMS documentaries between January 2016 and October 2019 and 20 Air Ambulance websites that had patient stories published until October 2020. In all, 628 patients identified fit the eligibility criteria: 311 from HEMS documentaries and 317 patients from Air Ambulance websites.

Results

In all, 0.64% (4/628) of patients died before the hospital, including 0.96% (3/311) of patients on HEMS documentaries and 0.32% (1/317) of patients on Air Ambulance websites. In addition, 2.23% (14/628) of patients died according to their final mention in the data source, including 1.93% (6/311) of patients on HEMS documentaries and 2.52% (8/317) of patients on Air Ambulance websites.

Conclusions

This study suggests under-reporting of poor patient outcomes in HEMS documentaries and on UK Air Ambulance websites. This could be attributed to the logistical and ethical implications of capturing and presenting poor outcomes but likely impacts upon public perception. Medical professionals should recognize this in order to proactively address potential misconceptions when communicating with patients and their families.

## Introduction

Helicopter emergency medical services (HEMS) are reliant on charitable donations to provide prehospital care to some of the sickest patients [1]. British HEMS documentaries, attracting millions of viewers [2], and publicly available patient stories on Air Ambulance websites are vital to raise awareness and funding for Air Ambulance charities. In all, 15.6% of HEMS patients do not survive due to the severity of their injuries [3]. The severity of injury and mortality in these patients, in the uncontrolled prehospital environment, pose further ethical and practical challenges to documentary makers and others involved in retelling the story [4,5]. These challenges may mean fewer patients with poor outcomes are presented to viewers, thereby contributing toward a bias in the outcomes presented.

Fictional medical dramas and news outlets often misrepresent patient health outcomes, introducing potential information bias to the public. Fictional medical programs present overinflated survival rates from CPR and optimistic outcomes regarding prognosis in comatose patients (defined as a period of unconsciousness lasting at least 24 hours) [6-8]. This has been thought to contribute toward unrealistic public expectations of recovery from CPR [9]. Differing expectations of recovery may lead to family disagreements about treatment during end-of-life decisions, as in the case of Schiavo [10]. News outlets also disproportionately report cases, overrepresenting deaths and traumatic injury in their reports [11]. This both skews public perception of outcomes from trauma and directly impacts upon survivors’ experiences following a distressing event [12].

Despite abundant research investigating how fictional programs and news outlets present patient health outcomes, there has not been a comprehensive study that investigates how HEMS documentaries or Air Ambulance websites present patient outcomes.

Objectives

The primary objective was to assess the frequency of reported poor outcomes (mortality) in the patient stories section of UK Air Ambulance websites and in documentaries focusing on HEMS. Secondary objectives were to record the mechanism of injury and injuries sustained, the degree of disability, and whether HEMS was reflected in a positive or negative light when death was portrayed.

## Materials and methods

This study is a two-armed retrospective cohort study looking at patient outcomes presented in publicly available HEMS documentaries and Patient Story (or equivalent) sections on websites of Air Ambulance charities.

Arm one

Publicly accessible (through free or paid-for screening services) HEMS documentaries released between January 2016 and October 2019 were screened. Five different documentary series fit the eligibility criteria (Table 1): Helicopter ER, Emergency Helicopter Medics, An Hour to Save Your Life, Trauma Doctors, and Air Ambulance ER. If multiple seasons were available, data was only collected from patients shown in the first two publicly available seasons screened chronologically to limit data skew.

**Table 1:** Eligibility criteria HEMS, helicopter emergency medical services

TV Shows		Websites	
Inclusion	Exclusion	Inclusion	Exclusion
TV series focusing on HEMS teams in the UK	TV series that include HEMS, but HEMS are not the main emergency service televised	Any form of media focusing on a patient story on any official UK Air Ambulance charity website	Cases found under the “news” section of the Air Ambulance website unless the patient stories are clearly separated within the news section
Broadcasted from April 2016 until October 2019	Broadcasted before April 2016 or after October 2019	Uploaded to the website until October 2020	Uploaded to the website after October 2020
Viewers see patient or scene	Job is canceled, and viewers do not see the patient	First of a series of stories talking about one case	Subsequent series of stories talking about the one case
Cases that focus on an individual patient (even if multiple patients are at scene)	Cases in which multiple patients are the focus	Cases that focus on an individual patient (even if multiple patients are at scene)	Cases in which multiple patients are the focus
Two first series from the date of inclusion	Third series of program and onward from date of inclusion		Inter-hospital patient transfer services
	Inter-hospital patient transfer services		

Arm two

UK-based Air Ambulance providers were identified using a national database, and their charity websites were accessed [13]. Twenty regional Air Ambulance organizations had published patient stories on their websites at the time of data collection and were included in the study. The 20 most recently published patient stories uploaded before October 2020 that fit the eligibility criteria (Table 1) from each website were included in the study. If multiple sections of the website focused on patient stories, then data collection was distributed evenly across each section.

Two data collectors (blinded to each other) independently reviewed each case, and data was assigned to pre-agreed categories. Data collectors then compared the two sets of data and resolved any discrepancies. When agreement could not be reached, a third blinded person provided the deciding categorization.

The following data was collected for both study arms: age (over 16 years to adult); gender of patient; medical or trauma case; mechanism of injury, injuries sustained, survival to hospital, and degree of disability according to the modified Glasgow outcome score (GOS); and how the provider was reflected if death occurred. Patients were followed up until the end of the episode or published patient stories.

Two categories

“Was a time frame between the incident and outcome given?” and “What was the time frame between the incident and outcome given?” were originally collected but later removed from the study to improve data clarity.

Additional data collected for HEMS programs included the program name and episode. Additional data collected from Air Ambulance websites included the website name and the media form in which the patient’s story was told (writing, podcast, and video).

Injuries sustained were categorized according to the books “Trauma Care: Beyond the Resuscitation Room” and “Trauma” [14,15]. Injuries were included in data collection if they were mentioned or were visually apparent from the data source, and the data collector felt they were either the focus of the case or clinically significant. There was no limit on the number of injuries that could be recorded for each patient, but where multiple injuries were sustained, only injuries deemed to be life-threatening or had the potential to significantly impact the patient’s life were recorded. For example, an “upper limb injury” would be recorded if an arm bruise was the only injury experienced by a patient, but not if the patient had multiple other injuries that were felt to be the episode’s focus. Conjectured but unconfirmed injuries (such as possible C-spine injury on initial assessment) were not included unless they were confirmed at a later point.

The degree of disability in neurological patients is usually assessed using the GOS [16]. The authors of this study assessed the degree of disability by using a modified version of the GOS, which was adapted to include physical descriptors of disability. A consensus decision was made by the working group to allocate a GOS to common phrases used by data sources to describe patient outcomes to ensure consistency throughout (Table 2). Patients were assigned a GOS according to their final mention in the data source. If death or disability later occurred, which appeared unrelated to the original incident, then the previous GOS was used. If a patient sustained multiple injuries but only one injury was mentioned by the data source when describing recovery, the GOS for the mentioned injury was used. It was assumed that the public would interpret that the patient had recovered well enough from their other injuries for them not to be mentioned.

**Table 2:** Modified Glasgow Outcome Scale

Scale	Category	Description	Patient descriptors
1	Death	As stated	N/A
2	Persistent vegetative state	Sleep/wake non-sentient	N/A
3	Severe disability	Conscious but dependant	In wheelchair; loss of sight; above the knee/elbow amputation
4	Moderate disability	Independent but disabled	“Mobility problems”; use of prosthesis, cast, and walking stick or sling; problems with vision (but not complete loss of sight); short-term memory loss; below the knee/elbow amputation
5	Good recovery	Resumption of normal life but may have mild neurological, physical, or psychological deficits	No mention of limitations/disabilities and evidence that they have resumed normal life (sitting in a chair does not suffice, but walking around does); mild symptoms may be mentioned; “back on his feet”; “back riding bike/back on the saddle”; back to previous activity; returned to work (previous job); “no serious injuries”
6	Undetermined	Unable to discern the level of disability	Only mentions future plans without any mention of current condition. “He plans to repair his motorbike and ride again”. Anything that refers to current process of recovering (recovering well/on the road to recovery)

Due to the low number of patient deaths, reliable quantitative analysis of how HEMS providers were portrayed following death was not possible, so qualitative data was presented instead.

Data was processed using Microsoft Excel 2016. Patient outcome data on HEMS programs and websites were presented as a whole and also according to each program and website individually. As there were no comparisons made between groups, no statistical tests of difference or descriptive statistics were appropriate to calculate.

According to the Joint Research Management Office (JRMO) guidance at the Queen Mary University of London, London, UK, ethical approval was not required for this study. Where possible, the study adhered to STROBE guidelines for reporting observational studies.

Patient and public involvement

Due to funding constraints, there was no patient or public involvement when setting the research question or outcome measures, and no patients were involved in the interpretation or writing up of results.

## Results

In Table 3, the main results are shown.

**Table 3:** Main results The data has been represented as N (%).

		Documentaries, n (%)	Websites, n (%)	Total, n (%)
Patient number		311	317	628
Demographics	Adult (>16 years)	279 (89.71%)	257 (81.07%)	536 (85.35%)
	Paediatric (<16 years)	32 (10.29%)	57 (17.98%)	89 (14.17%)
	Unknown	0 (0.00%)	3 (0.95%)	3 (0.48%)
	Male	234 (75.24%)	200 (63.09%)	434 (69.11%)
Mechanism	Trauma (total)	262 (84.24%)	243 (76.66%)	505 (80.41%)
	Road traffic collision	117 (37.62%)	134 (42.27%)	251 (39.97%)
	Fall from height (>2 m)	40 (12.86%)	20 (6.31%)	60 (9.55%)
	Fall (<2 m)	54 (17.26%)	38 (11.99%)	92 (14.65%)
	Assault (gunshot)	0 (0.00%)	0 (0.00%)	0 (0.00%)
	Assault (stabbing/penetrating injury)	7 (2.25%)	3 (0.95%)	10 (1.59%)
	Assault (other)	0 (0.00%)	1 (0.32%)	1 (0.16%)
	Trauma from animal	14 (4.50%)	15 (4.73%)	29 (4.62%)
	Under train	0 (0.00%)	0 (0.00%)	0 (0.00%
	Self-harm	0 (0.00%)	0 (0.00%)	0 (0.00%)
	Drowning/near drowning	1 (0.32%)	1 (0.32%)	2 (0.32%)
	Other trauma	29 (9.32%)	31 (9.78%)	60 (9.55%)
	Medical (total)	49 (16.08%)	74 (23.34%)	123 (19.59%)
Injury or illness	Head injury	66 (21.22%)	98 (30.91%)	164 (26.11%)
	Spinal injury	77 (24.76%)	57 (17.98%)	134 (21.34%)
	Abdominal trauma	24 (7.72%)	28 (8.83%)	52 (8.28%)
	Chest trauma	62 (19.94%)	65 (20.50%)	127 (20.22%)
	Pelvic injury	28 (9.00%)	48 (15.14%)	76 (12.10%)
	Upper limb injury	49 (15.76%)	60 (18.93%)	109 (17.36%)
	Lower limb injury	88 (28.30%)	112 (35.33%)	200 (31.85%)
	Facial injury	16 (5.14%)	28 (8.83%)	28 (4.46%)
	Skin injuries	14 (4.50%)	3 (0.95%)	17 (2.71%)
	Burns	5 (1.61%)	7 (2.21%)	12 (1.91%)
	Drowning/diving injuries	0 (0.00%)	3 (0.95%)	3 (0.48%)
	Electrical injuries	0 (0.00%)	0 (0.00%)	0 (0.00%)
	Hypothermia	0 (0.00%)	3 (0.95%)	3 (0.48%)
	Medical respiratory emergency	7 (2.25%)	8 (2.52%)	15 (2.39%)
	Medical cardiovascular emergency	24 (7.72%)	43 (13.56%)	67 (10.67%)
	Medical vascular emergency	2 (0.64%)	2 (0.63%)	4 (0.64%)
	Medical cerebrovascular emergency	5 (1.61%)	7 (2.21%)	12 (1.91%)
	Medical childbirth-related emergency	0 (0.00%)	2 (0.63%)	2 (0.32%)
	Other medical	13 (4.18%)	12 (3.79%)	25 (3.98%)
	Other trauma	4 (1.29%)	11 (3.47%)	15 (2.39%)
Survival to hospital	Yes	308 (99.04%)	314 (99.05%)	622 (99.04%)
	No	3 (0.96%)	1 (0.32%)	4 (0.64%)
	Unknown	0 (0.00%)	2 (0.63%)	2 (0.32%)
Outcome	GOS 1 (death)	6 (1.93%)	8 (2.52%)	14 (2.23%)
	GOS 2 (persistent vegetative state)	0 (0.00%)	0 (0.00%)	0 (0.00%)
	GOS 3 (severe disability)	13 (4.18%)	13 (4.10%)	26 (4.14%)
	GOS 4 (moderate disability)	28 (9.00%)	47 (14.83%)	75 (11.94%)
	GOS 5 (low/no disability)	167 (53.70%)	195 (61.51%)	362 (57.64%)
	GOS 6 (Undetermined)	97 (31.19%)	54 (17.03%)	151 (24.04%)
How was service portrayed when death occurred?	Total deaths	6	8	14
	Positively	2 (33.33%)	8 (2.52%)	10 (71.43%)
	Negatively	0 (0.00%)	0 (0.00%)	0 (0.00%)
	Neutral/mixed	3 (50.00%)	0 (0.00%)	3 (21.43%)
	Undetermined	1 (16.67%)	0 (0.00%)	1 (7.14%)

In all, 628 patients fit the eligibility criteria: 311 from HEMS documentaries and 317 patients from Air Ambulance websites. In addition, 536/628 (85.35%) patients were adults: 279/311 (89.71%) from HEMS documentaries and 257/317 (81.07%) from Air Ambulance websites. Also, 434/628 (69.11%) patients were male: 234/311 (75.24%) from HEMS documentaries and 200/317 (63.09%) from Air Ambulance websites.

Thus, 622/628 (99.04%) patients survived until hospital admission. The survival rate to hospital was equally high across both documentaries (308/311; 99.04%) and websites (314/317; 99.05%).

A total of 362/628 (57.64%) patients had no/low disability (GOS 5) according to their final mention in the data source, making it the most common GOS across HEMS documentaries (167/311; 53.70%) and Air Ambulance websites (195/317; 61.51%). In all, 75/628 (11.94%) patients had moderate disability (GOS 4), accounting for 9.00% (28/311) of HEMS documentaries outcomes and 14.83% (47/317) of Air Ambulance website outcomes. Severe disability (GOS 3) made up a small minority of cases (26/628; 4.14%), while there were no portrayals of patients in a persistent vegetative state (GOS 2). In addition, 14/628 (2.23%) patients died (GOS 1), with slightly less deaths in HEMS documentaries (6/311; 1.93%) compared to Air Ambulance websites (8/317; 2.52%). Thus, 151/628 (24.04%) patients were allocated GOS 6 (undetermined outcome), which varied across HEMS documentaries (97/311; 31.19%) and Air Ambulance websites (54/317; 17.03%). When undetermined outcomes were re-allocated as GOS 3 or above (undetermined was always allocated due to GOS 3, 4, and 5 being indistinguishable from the information provided), 614/628 (97.77%) of patients were GOS 3 or higher.

Trauma presentations accounted for most cases in both HEMS documentaries (262/311 84.24%) and Air Ambulance websites (243/317; 76.66%). Road traffic collision (RTC) was the most common mechanism of injury across HEMS documentaries (117/311; 37.62%) and Air Ambulance (134/317; 42.27%) websites.

Tables 4, 5 display key outcome data according to each HEMS documentary and website.

**Table 4:** Key outcome data according to each Air Ambulance charity website *GOS 6 = undetermined The data has been represented as N (%). GOS, Glasgow Outcome Score

Website	Number of patients per website, n (% of total patients)	Survival to hospital, n (% of patients from website)		GOS, n (% of patients from website)					
		Yes	No/unknown	1	2	3	4	5	6*
Charity 1	20 (6.31%)	20 (100.00%)	0 (0.00%)	0 (0.00%)	0 (0.00%)	1 (5.00%)	2 (10.00%)	13 (65.00%)	4 (20.00%)
Charity 2	20 (6.31%)	20 (100.00%)	0 (0.00%)	1 (5.00%)	0 (0.00%)	1 (5.00%)	2 (10.00%)	11 (55.00%)	5 (25.00%)
Charity 3	20 (6.31%)	20 (100.00%)	0 (0.00%)	0 (0.00%)	0 (0.00%)	0 (0.00%)	9 (45.00%)	7 (35.00%)	4 (20.00%)
Charity 4	20 (6.31%)	18 (90.00%)	2 (10.00%)	2 (10.00%)	0 (0.00%)	0 (0.00%)	2 (10.00%)	12 (60.00%)	4 (20.00%)
Charity 5	20 (6.31%)	19 (95.00%)	1 (5.00%)	1 (5.00%)	0 (0.00%)	1 (5.00%)	5 (25.00%)	12 (60.00%)	1 (5.00%)
Charity 6	15 (4.73%)	15 (100.00%)	0 (0.00%)	0 (0.00%)	0 (0.00%)	1 (6.67%)	0 (0.00%)	13 (86.67%)	1 (6.67%)
Charity 7	5 (1.58%)	5 (100.00%)	0 (0.00%)	0 (0.00%)	0 (0.00%)	2 (40.00%)	1 (20.00%)	2 (40.00%)	0 (0.00%)
Charity 8	20 (6.31%)	20 (100.00%)	0 (0.00%)	1 (5.00%)	0 (0.00%)	1 (5.00%)	1 (5.00%)	16 (80.00%)	1 (5.00%)
Charity 9	20 (6.31%)	20 (100.00%)	0 (0.00%)	0 (0.00%)	0 (0.00%)	0 (0.00%)	3 (15.00%)	14 (70.00%)	3 (15.00%)
Charity 10	16 (5.05%)	16 (100.00%)	0 (0.00%)	0 (0.00%)	0 (0.00%)	0 (0.00%)	3 (18.75%)	12 (75.00%)	1 (6.25%)
Charity 11	16 (5.05%)	16 (100.00%)	0 (0.00%)	2 (12.50%)	0 (0.00%)	0 (0.00%)	2 (12.50%)	7 (43.75%)	5 (31.25%)
Charity 12	20 (6.31%)	20 (100.00%)	0 (0.00%)	1 (5.00%)	0 (0.00%)	5 (25.00%)	3 (15.00%)	10 (50.00%)	1 (5.00%)
Charity 13	20 (6.31%)	20 (100.00%)	0 (0.00%)	0 (0.00%)	0 (0.00%)	0 (0.00%)	0 (0.00%)	19 (95.00%)	1 (5.00%)
Charity 14	20 (6.31%)	20 (100.00%)	0 (0.00%)	0 (0.00%)	0 (0.00%)	1 (5.00%)	8 (40.00%)	10 (50.00%)	1 (5.00%)
Charity 15	3 (0.95%)	3 (100.00%)	0 (0.00%)	0 (0.00%)	0 (0.00%)	0 (0.00%)	0 (0.00%)	1 (33.33%)	2 (66.67%)
Charity 16	12 (3.79%)	12 100.00%)	0 (0.00%)	0 (0.00%)	0 (0.00%)	0 (0.00%)	2 (16.67%)	2 (16.67%)	8 (66.67%)
Charity 17	4 (1.26%)	4 (100.00%)	0 (0.00%)	0 (0.00%)	0 (0.00%)	0 (0.00%)	0 (0.00%)	4 (100.00%)	0 (0.00%)
Charity 18	6 (1.89%)	6 (100.00%)	0 (0.00%)	0 (0.00%)	0 (0.00%)	0 (0.00%)	1 (16.67%)	4 (66.67%)	1 (16.67%)
Charity 19	20 (6.31%)	20 (100.00%)	0 (0.00%)	0 (0.00%)	0 (0.00%)	0 (0.00%)	1 (5.00%)	16 (80.00%)	3 (15.00%)
Charity 20	20 (6.31%)	20 (100.00%)	0 (0.00%)	0 (0.00%)	0 (0.00%)	0 (0.00%)	2 (10.00%)	10 (50.00%)	8 (40.00%)
Total	317	314 (99.05%)	3 (0.95%)	8 (2.52%)	0 (0.00%)	13 (4.10%)	47 (14.83%)	195 (61.51%)	54 (17.03%)

**Table 5:** Key outcome data according to each HEMS Documentary ^*^GOS 6 = undetermined The data has been represented as N (%). GOS, Glasgow Outcome Score; HEMS, helicopter emergency medical services; EHM, emergency helicopter medics; AHTSYL, An Hour To Save Your Life

HEMS documentary	Number of patents per program, n (% of total patients)	Survival to hospital, n (% of patients from program)		GOS, n (% of patients from program)					
		Yes	No	1	2	3	4	5	6^*^
Helicopter ER	176 (56.59%)	174 (98.86%)	2 (1.14%)	2 (1.14%)	0 (0.00%)	6 (3.41%)	15 (8.52%)	98 (55.68%)	55 (31.25%)
EHM	76 (24.44%)	75 (98.68%)	1 (1.32%)	3 (3.95%)	0 (0.00%)	3 (3.95%)	3 (3.95%)	33 (43.42%)	34 (44.74%)
AHTSYL	12 (3.86%)	12 (100.00%)	0 (0.00%)	0 (0.00%)	0 (0.00%)	4 (33.33%)	2 (16.67%)	5 (41.67%)	1 (8.33%)
Trauma doctors	24 (7.72%)	24 (100.00%)	0 (0.00%)	1 (4.17%)	0 (0.00%)	0 (0.00%)	5 (20.83%)	15 (62.50%)	3 (12.50%)
Air Ambulance ER	23 (7.40%)	23 (100.00%)	0 (0.00%)	0 (0.00%)	0 (0.00%)	0 (0.00%)	3 (13.04%)	16 (69.57%)	4 (17.39%)
Total	311	308 (99.04%)	3 (0.96%)	6 (1.93%)	0 (0.00%)	13 (4.18%)	28 (9.00%)	167 (53.70%)	97 (31.19%)

When deaths occurred, HEMS was consistently portrayed in a positive light (defined as a direct comment praising staff, the service, or timings) both in programs and on websites. Families reported common themes, including the “empathy” or “compassion” of medical personnel, the quality of medical treatment received, and the support offered during and after the event. Families repeatedly described being grateful for aftercare provided by HEMS, which provided explanations and closure after a sudden event. There was also repeated emphasis on HEMS-delivered treatment, meaning that families had time to say goodbye.

## Discussion

Our results found that 1.93% (6/311) of patients on HEMS programs and 2.52% (8/317) of patients on Air Ambulance websites died. This is observed to be markedly less than the results of two UK studies, the first of which found that 12.5% (35/281) of patients treated by HEMS died before hospital discharge, and the second found that 11.7% (48/412) died within 30 days [17,18]. A further study using the TARN database found total mortality to be 7% from a sample size of 307,307 patients [19]. Regional Air Ambulance data also presents mortality as higher than the outcomes of this study [20]. Poor patient outcomes are therefore under-represented in HEMS documentaries and on the Patient Stories section of UK Air Ambulance websites. The positive outcome bias apparent in this study is likely due to both logistical and ethical implications of portraying negative outcomes. 

Reasons for positive outcome bias

The time-critical nature of HEMS medical treatment and the vulnerability of unwell patients means that patient consent for media portrayals is usually gained retrospectively. This leads to ethical problems for gaining consent in cases where patients do not survive [5], and asking for consent from bereaved families is not always appropriate. The BBC’s editorial guidelines specify “weighing up the public interest in the story with any distress an approach may cause the family” and that they “would normally only broadcast the footage with the family’s clear provable consent” [21]. However, deaths were included in programs and on Air Ambulance websites, thereby demonstrating a strong relationship between families and the organizations.

Producers may also be wary of the psychological impact on families of unexpectedly seeing a deceased loved one portrayed in the media or of reliving the events leading to their death. The risk of re-traumatizing families is particularly a problem for TV shows, given their visual nature and real-time portrayal of events. Repeated screenings may exacerbate the risk of re-traumatization, since repeats may occur many months or even years after consent is given.

A particular problem for TV shows arises due to the sensitivities around portraying dead bodies and the need to maintain dignity. In one case, a deceased patient was submerged underwater in their car. While the extrication attempt was shown, viewers never saw the patient’s body. It was felt that the producer’s decision was ethically correct as to have shown the body would have been intrusive, contrary to patient dignity, and narratively unnecessary.

Many of these TV shows are broadcast before or shortly after the watershed (the time after which content unsuitable for children can be broadcast). Although often shown after the watershed, Ofcom (the regulatory authority for television broadcasting in the UK) states that “the transition to more adult material must not be unduly abrupt at the watershed… strongest material should appear later in the schedule” [22]. Similarly, it states that violence and its after-effects should be limited before the watershed. Therefore, particularly graphic scenes or upsetting narratives may consequently be avoided. Practically, it is also possible that criminal behaviors resulting in injury (for example, stabbings and shootings) may not have been filmed due to additional risk to personnel, thereby skewing data.

Survival bias also plays a role in selecting publicly available cases. If a patient is dead on arrival or dies on-scene, there may be less medical treatment provided. Websites aim to educate the public on HEMS interventions, and TV shows require substantial footage, so both may be more likely to select cases where patient journeys are followed for a significant amount of time. Websites also aim to fundraise money for HEMS missions, and positive outcomes of their interventions may encourage donations.

Impact of positive outcome bias

While there are clear reasons for positive outcome bias to exist, it is important that we recognize the impact. Most problematically, this bias may lead to unrealistic expectations about the outcomes of individuals whose level of risk necessitates HEMS treatment. As a result, patient families may be unprepared for more negative outcomes that may occur despite the best treatment. Particularly given the unexpected and sudden nature of many HEMS callouts, this lack of emotional preparation may make it difficult for families to process grief. 

The wider public may also feel falsely reassured about the ability of medical personnel to successfully treat serious injury. A more realistic portrayal of outcomes might help with injury prevention, for example, by encouraging seatbelt and helmet use. Positive bias also risks underestimating and downplaying the “moral injury” suffered by responders to traumatic events [23], leading to less support for first responders.

Our findings tie into a broader debate about the interaction between emergency medicine and journalism, which has been given particular urgency by the huge popularity of ambulance and hospital TV shows, such as 24 Hours in A&E and Ambulance [24-26]. Part of the problem is due to the dual aims of these shows, to both educate and entertain. As such, there is a risk that the reader or viewer becomes a voyeur, watching others’ suffering for entertainment. A balance must be struck between the potential harm to individual patients and the benefits brought through public education.

Future change

Increasing the frequency of portrayals of poor outcomes in both TV shows and websites would provide a more realistic picture. This may not always be possible, so producers and websites could include a disclaimer to acknowledge outcomes that may not be representative of real-life data.

While poor outcomes were few, media portrayals of poor outcomes were done sensitively, balancing public education with patient dignity and privacy. Poor outcomes that were directly reflected upon by family members returned control to families, who were able to choose how their relatives were portrayed. In addition, health care personnel often emphasized the wider context of individual cases, countering the bias toward positive outcomes on a bigger scale.

In terms of attracting funding, we found that by portraying poor outcomes, attention could be brought to HEMS services such as “aftercare” that the public might not otherwise know about.

These successes can be implemented on a broader scale, and in particular, there is a real possibility of improving the reporting of bereavement on websites. HEMS providers have direct control over their websites, meaning content changes can be made quickly and inexpensively. Moreover, the less visual formats of websites mean several of the problems of TV shows are avoided. There is less risk of patient families unexpectedly seeing stories about deceased family members, and patient dignity is easier to maintain. Changes in tone can be easily implemented by slight changes in wording.

Already, there have been recent attempts by HEMS providers to challenge this bias. Recognizing the disconnect in public expectations of HEMS outcomes, Child Bereavement UK has been working with LAA to support families and health care providers better [27]. As part of this culture change, LAA has introduced new training for clinical staff and produced a series of podcasts that include cases with poor outcomes. The extended podcast format allows an exploration of the impact of bereavement on families, as well as signposting others in similar situations to support [28].

Even if media portrayals of HEMS continue to be biased toward positive outcomes, recognizing this fact is vital in shaping how we interact with patients and their families. Much like CPR, which has been misrepresented in media portrayals [6,7], by recognizing the impact of these misrepresentations on the public, medical personnel can be better prepared to challenge misconceptions when communicating with a patient’s family. Future research should focus on capturing public perception of the likelihood of survival in HEMS patients.

By recognizing the bias toward positive outcomes in portrayals of HEMS operations, we can likewise better shape how families experience bereavement. Prehospitally, this means explaining HEMS interventions to families and signposting possible outcomes. At the hospital, this means providing targeted support when breaking bad news. Additionally, as is increasingly being done by HEMS teams, it means providing aftercare to families. In doing so, we can support families better both during and after HEMS medical interventions.

Limitations of study

The study design may have led to an under-representation of mortality in the data. Cases with more than one patient were excluded from data collection. Multi-casualty incidents may have higher impact mechanisms and lower survival rates, leading to a disproportionate representation of mortality in the study. Cases where patients died before HEMS providers arrived and the referral was canceled were also not included.

Data collectors had a medical background, so they may have interpreted injury and functionality differently from the general public, especially as comments about disability on websites and programs were often ambiguous. Visible cues and pictures were used to determine GOS, likely resulting in an overestimation of the impact of physical deficits and an under-representation of the impact of mental health problems. Relying on visible cues and comments also has limitations when trying to assess disability reliably. The modified GOS used has not been validated for patients without head injury. 

Only programs with HEMS cases as the sole focus were included. HEMS cases also feature on shows such as “24 Hours in A&E,” so the data presented may not be representative of the public perception of HEMS.

## Conclusions

This study found lower death rates presented on HEMS documentaries and UK Air Ambulance websites compared to prior studies. This suggests under-reporting of poor outcomes by these agencies, which could be attributed to the logistical and ethical implications of capturing and presenting poor outcomes to the public. Under-representation of poor outcomes may lead to the public forming unrealistic expectations of outcomes in HEMS patients. By recognizing the impact of these misrepresentations on the public, medical personnel can be better prepared to challenge misconceptions when communicating with patients and their families.
